# Jet fuel exposure and auditory outcomes in Australian air force personnel

**DOI:** 10.1186/s12889-019-7038-0

**Published:** 2019-05-31

**Authors:** Adrian Fuente, Louise Hickson, Thais C. Morata, Warwick Williams, Asaduzzaman Khan, Eduardo Fuentes-Lopez

**Affiliations:** 1 School of Health and Rehabilitation Sciences, The University of Queensland, Brisbane, Australia; 2 Centre de recherche de l’Institut universitaire de gériatrie de Montréal, Montreal, Quebec, Canada; 3 National Institute for Occupational Safety and Health, Cincinnati, OH USA; 4 National Acoustic Laboratories, Sydney, NSW Australia; 5 Carrera de Fonoaudiología, Departamento de Ciencias de la Salud, Pontificia Universidad Católica de Chile, Santiago, Chile

**Keywords:** Exposure, Hearing loss, Jet fuels, Military personnel, Noise

## Abstract

**Background:**

Animal data suggest that jet fuels such as JP-8 are associated with hearing deficits when combined with noise and that the effect is more pronounced than with noise exposure alone. Some studies suggest peripheral dysfunction while others suggest central auditory dysfunction. Human data are limited in this regard. The aim of this study was to investigate the possible chronic adverse effects of JP-8 combined with noise exposure on the peripheral and central auditory systems in humans.

**Methods:**

Fifty-seven participants who were current personnel from the Royal Australian Air Force were selected. Based on their levels of exposure to jet fuels, participants were divided into three exposure groups (low, moderate, high). Groups were also categorised based on their noise exposure levels (low, moderate, high). All participants were evaluated by tympanometry, pure-tone audiometry (1–12 kHz), distortion product otoacoustic emissions (DPOAEs), auditory brainstem response (ABR), words-in-noise, compressed speech, dichotic digit test, pitch pattern sequence test, duration pattern sequence test and adaptive test of temporal resolution. All auditory tests were carried out after the participants were away from the Air Force base for a minimum of two weeks, thus two weeks without jet fuel and noise exposure.

**Results:**

Jet fuel exposure was significantly associated with hearing thresholds at 4 and 8 kHz; average hearing thresholds across frequencies in the better ear; DPOAEs at 2.8, 4 and 6 kHz; ABR wave V latency in the right ear; compressed speech and words-in-noise. Further analyses revealed that participants with low exposure level to jet fuels showed significantly better results for the aforementioned procedures than participants with moderate and high exposure levels. All results were controlled for the covariates of age and noise exposure levels.

**Conclusions:**

The results suggest that jet fuel exposure, when combined with noise exposure, has an adverse effect on audibility in humans. Taking all the test results into consideration, jet fuel exposure combined with noise exposure specifically seems to affect the peripheral hearing system in humans.

## Background

Hydrocarbon jet fuels are components used to power jet airplanes [1]. They are found in jet propulsion fuels such as JP-4, JP-5, JP-7, JP-8, gasoline, diesel fuels and kerosene [2]. They are made of long- and short-chain aromatic and aliphatic hydrocarbons [1] and are among the most common occupational chemical exposures encountered by military and civilian workers [3].

In the North Atlantic Treaty Organization (NATO) countries, JP-8 is the most standard jet fuel utilised for military purposes. About 6 billion gallons of JP-8 are used every year [4]. JP-8 is less toxic and safer than JP-4 because it contains lower percentages of ototoxicants such as toluene and xylene [5]. However, JP-8 still contains many ototoxic aromatic hydrocarbons.

Environmental exposure to jet fuels has been associated with several health conditions, such as immune system dysfunction, neurobehavioural problems, developmental/reproductive dysfunction and hepatic, pulmonary and renal dysfunction [4, 6]. Recently, vestibular dysfunction has been associated with jet fuel exposure [7, 8]. In addition, JP-4 and JP-8 have been associated with peripheral [9, 10] and central auditory nervous system [2] dysfunctions in the animal model.

Fechter et al. [9] found that a single exposure to JP-8 (1000 mg/m^3^) did not affect the outer hair cell (OHC) function as opposed to recurrent exposure at the same level for a period of 5 days. A 20 dB decrease in distortion product otoacoustic emissions (DPOAE) between 8 and 12 kHz that slightly recovered after 4 weeks was observed. This effect on OHCs was more pronounced when rats were simultaneously exposed to JP-8 and noise than when they were exposed to noise alone. Later, Fechter et al. [10] found an adverse effect of JP-8 only when combined with noise on DPOAE in experimental animals. However, no decrement in hearing thresholds or increase in OHC loss was observed. In another study carried out by Fechter et al. [11], rats were simultaneously exposed to JP-8 and noise for a longer period of time each day than in the previous study, for 4 weeks (5 days/week). No additional effect of JP-8 was observed on OHC. However, a greater hearing threshold shift for high frequencies (8–20 kHz), as measured by compound action potential, was observed in rats simultaneously exposed to JP-8 and noise than in rats exposed to noise alone. More recently, Guthrie et al. [2, 12] conducted two studies using auditory brainstem response (ABR) and DPOAE. In each study, a different strain of rats (Long-Evans, Fisher 344) were exposed to JP-8 and noise. An effect of JP-8 was observed on the central auditory nervous system by comparing the ABR amplitude for waves I, II and III. This effect was more pronounced when JP-8 was combined with noise. No effect of JP-8 on the peripheral auditory system, as evaluated by DPOAE, was observed.

In humans, Kaufman et al. [5] conducted a study with U.S. Air Force employees exposed to JP-4 and noise (> 85 dB and < 95 dB). The results showed that chronic exposure to noise (> 85 dBA) and JP-4 increases the odds of developing permanent hearing loss. However, exposure to JP-4 alone did not show an effect on pure-tone thresholds.

Thus, based on animal data, it may be hypothesised that workers such as aviation personnel who are exposed to JP-8, may exhibit poorer hearing thresholds than nonexposed populations along with signs of either peripheral or central auditory dysfunction. Therefore, the aim of this study was to investigate the possible chronic adverse effects of JP-8 exposure on the peripheral and central auditory systems in humans.

## Methods

### Study design

This is a cross-sectional study of Royal Australian Air Force (RAAF) personnel exposed to different levels of jet fuels and noise.

### Ethical approval

All research procedures were approved prior to the commencement of the study by the University of Queensland’s Human Research Ethics Committee and by the Australian Defence Human Research Ethics Committee.

### Study participants and data collection

Participants exposed to jet fuels were selected using a nonprobability, convenience sampling technique. Research participants were personnel from a base of the RAAF located in Queensland, Australia. Around 5000 people work at this airbase. All personnel were invited to take part in the research. Two visits were carried out to invite prospective participants by providing oral and written information about the project. An email address and telephone number were provided for the prospective participants to contact the research team in case they wanted to participate in the study. Initial inclusion criteria were (a) being in defence for at least 1 year and (b) age between 18 and 64 years.

Each participant who contacted the research team and decided to take part in the study was individually scheduled for a 120-min appointment at the audiology clinic of the University of Queensland. All participants attended the appointment after a minimum of 2 weeks away from the base without being exposed to jet fuels and noise. Two weeks away from the base was considered as the minimum period in order to control for acute effects of jet fuels on the auditory system (Moen et al. [13]). A trained audiologist conducted all audiological procedures. An informed consent form was provided and participants were asked to sign it if they agreed to proceed with the assessments. Then, a medical and occupational history questionnaire was carried out. The aim of this questionnaire was to select participants with an absence of medical conditions associated with auditory disorders and to determine noise-exposure levels based on self-report. After the interview, bilateral otoscopy (mini Heine 2000, Herrsching, Germany) and tympanometry (Otometrics, Madsen Zodiac 901, Taastrup, Denmark) were conducted. Only participants with normal otoscopy and normal middle ear function (tympanic peak pressure between − 100 and + 50 daPa and static compliance ≥0.3 mL) [14] were included in the sample. Participants were then evaluated with pure-tone audiometry, distortion product otoacoustic emissions (DPOAE), auditory brainstem response (ABR) and psychoacoustic tasks to evaluate central auditory functions such as temporal processing, dichotic listening and auditory closure. The order of testing was the same for all participants.

### Workplace environment

Exposure to chemicals among those who work at the studied airbase includes agents such as jet fuels (i.e. jP-8), organic solvents (e.g. toluene, xylene) and other chemicals. Occupational exposure to jet fuels can occur during refuelling and defuelling operations, cold engine starts and during mechanical activities. The use of solvents include cleaning, degreasing, vehicle maintenance and repair, paint stripping and thinning oil-based paints. Some personnel have been exposed in more specific settings such as the RAAF F-111 Deseal/Reseal programs (DSRS). Chemical exposure may occur through the inhalation (aerosolised or vaporised fuel), dermal and/or oral routes of exposure, although the oral route is unusual. Personnel at the base are exposed to noise from aircraft movements to a varying degree. In addition, personnel are exposed to noise sources specific to their jobs.

### Exposure classifications

A priori jet fuel exposure groups (low, moderate, high) were assigned to the workers selected to participate in the study based on a combination of the following: (a) task group and task group history, taking into account current and past job category/mustering, (b) self-reported exposure level for each task group, (c) findings of multiple previous exposure assessment evaluations by independent contractors [15–17], and (d) expert evaluation by an occupational hygienist in RAAF. The selection of hazards for assessment by monitoring has been based on judgement of the nature of the hazard (e.g. toxicity of a chemical, level of noise, etc.) combined with exposure duration and frequency. Consequently, locations or job categories that were considered to be free of risks have not been evaluated, and that includes some of the participants of this study. Therefore, they were assigned to the low exposure group, unless they had a history of higher exposures in the past.

Higher weighting was given to exposure history prior to the 2001 F-111 Deseal/Reseal Board of Inquiry, and particularly for exposures during the 1970s and 1980s, when exposure protection was more likely to be deficient [18]. A 2010 industrial hygiene report [16] provided to the authors, stated that “the level of control of chemical substances on the base was observed to be excellent. Procedures were in place requiring personal protective equipment for all areas where chemicals were used. Most jobs where significant exposures would be expected (e.g. fuel tank entry, use of two pack products) follow strict procedures requiring air supplied positive pressure respiratory protection and full skin protection.” Volatile organic compounds were reported to be used in small quantities for relatively short durations in a variety of tasks and locations. The report also stated that while there was no significant exposure risk from the chemicals individually, in some areas, a cumulative exposure risk was possible. Table 1 provides examples of job categories for each jet fuel exposure group (i.e. low, moderate, high).

**Table 1:** Demographics and job categories for the three jet fuel exposure groups

	Jet fuel exposure group		
Variable	Low (*n* = 18)	Moderate (*n* = 15)	High (*n* = 24)
Age, years	42.3 (range: 25–60)	37.8 (range: 23–64)	38.7 (range: 24–55)
Gender	Female = 6	Female = 1	Female = 0
	Male = 12	Male = 14	Male = 24
Tenure in defence, years	17.8, 10.3 (5–41)	14.8, 13.0 (3–40)	19.7, 9.9 (5–38)
Job categories	Cook, training system officer, environmental health officer, air surveillance operator, information manager, administration, pilot, air traffic control, air refuelling operator, electrical engineer, vehicle administration, aircraft maintainer, firefighter	Warrant officer, avionics instructor, instructor, flighty sergeant, staff officer capability level, logistics, movements, aviation refueller, GSE fitter mechanic, GSE fitter mechanic trainee, technician, avionics technician	Aircraft technician, GT-1 armtech sergeant, air traffic controller, movements, airfield operations support, ops clerk, GSE fitter mechanic, avionics technician, loadmaster, structures technician, weapons/explosives technician, petroleum manager, safety advisor,

Exposure groups are based on participant exposure levels to jet fuels and chemicals and do not necessarily represent the same exposure category for noise

Similarly, a priori noise exposure categories (low, moderate, high) were assigned to the workers for each unit/area based on a comparison of historic records of noise measurements, conducted internally at the RAAF base, or under independent contracts with the National Acoustic Laboratories in the 1990’s, and with Vipac Consultants in 2011, and the noise exposure questions included in the initial questionnaire used in this study. These questions enquired about whether the person was currently exposed to occupational noise, and if so, the number of hours of exposure per week. In addition, questions included noise exposure in previous jobs and the number of hours exposed to noise. Job categories were also considered when classifying workers to a noise exposure category (i.e. low, moderate, high). Most exposures were considered to be low, with a few occasions of exposures of high intensity, for which hearing protection was required. Several types of hearing protectors were available across locations. Staff situated in areas close to the flightline such as firefighting staff and point of disembarkation hangar workers are exposed to the highest noise levels. This is due predominantly to aircraft activity, but with significant contribution from high-noise vehicles and equipment as well. According to records, noise dosimetry for firefighters ranged between 76 and 86 dB A-weighted Equivalent Sound Level (LAeq), and between 83 and 86 dB LAeq for point of disembarkation hangar workers. Another area of concern for noise exposure is the Number 6 Squadron (6SQN) that is a training and bomber squadron. Noise sources at the 6SQN Workshop include machinery (cold saws, grinders, drills, lathes, guillotines and milling machines) and aircraft. Noise dosimetry records revealed LAeq between 85 and 89 dB. Another area of concern for noise exposure is the Mechanical Equipment Operations Management Systems (MEOMS). The MEOMS workshop services a wide range of vehicles and equipment including tactical vehicles (e.g. bushmasters), fire trucks (e.g. panthers) and ground support equipment such as trailers. The three main sources of noise include aircraft flyovers, vehicle operation and workshop equipment. Noise dosimetry for MEOMS workshop staff ranged between 69 and 83 dB LAeq. Another area is the MEOMS-fuel equipment management systems. Two noise exposure groups are identified in this area, workshop staff and transport staff. Noise dosimetry revealed noise exposure levels between 79 and 84 dB LAeq for workshop staff. It is necessary to take into account that the participants’ exposure to noise, as well as chemicals was not daily, consistent or regular, which precludes a precise exposure estimation.

### Audiological assessment

#### Audiometric thresholds

Pure-tone air- and bone-conduction thresholds were obtained using an Orbiter 922 version 2 clinical audiometer (Madsen Electronics, Taastrup, Denmark) with TDH-39P headphones for frequencies between 0.5 to 8 kHz, and with Sennheiser HD 200 circumaural earphones (Sennheiser Co, Germany) for 10 and 12 kHz. A Radioear B-71 bone vibrator was used to obtain bone-conduction thresholds (1–4 kHz). Participants with the presence of an air-bone gap at two or more frequencies equal to or higher than 10 dB HL were excluded from the final sample.

#### Otoacoustic emissions (OAEs)

A DP Echoport (Otodynamics model ILO292, USB interference, Hatfield, England) was utilised for DPOAEs. This equipment was connected to a desktop computer that had ILO 292 OAE analysis software. The geometric means of f1 and f2 at 1, 1.4, 2, 2.8, 4, 6, and 8 kHz were used with primary levels (L1/L2) of 65/55 dB SPL and a primary ratio (f2/f1) of 1.22. Levels of the 2f1-f2 DPOAEs and the noise floor were registered as a function of f2. DPOAEs were expressed in dB signal-to-noise ratio (dB SNR).

#### Auditory brainstem response (ABR)

The ABR was recorded utilising Biologic Navigator Pro ABR equipment connected to an HP Compaq 6730b laptop computer. AgCI-AgCI electrodes were placed at the vertex (Cz, noninverting), ipsilateral mastoid (A1/A2, inverting) and forehead (Fpz, ground). Two recordings were obtained (2000 sweeps each) per ear using 80-dBnHL rarefaction click stimuli (27.7/s). Stimuli were presented monaurally.

#### Speech perception and central auditory function

For the behavioural assessment of central auditory function, a Pioneer DVD player DV 300 (Tokyo, Japan), connected to the audiometer mentioned above, was used. The following central auditory function procedures were carried out:

##### Compressed speech [19]

This test is part of the tonal and speech materials for auditory perceptual assessment, Disk 2.0, Auditory Research Laboratory, Veterans Affairs (VA) Medical Center, Mountain Home, Tennessee. A total of 50 monosyllabic words reduced 65% in their length with a 0.3 s reverberation were monaurally presented at 50 dB SL (according to the average of the pure-tone thresholds at 0.5, 1 and 2 kHz). The participant was asked to repeat back each word as it was heard. The average score between the right and left ears was obtained for analysis purposes ([right ear score (%) + left ear score (%)]/2).

##### Words-in-noise [20]

This test is part of the speech recognition and identification materials, Disk 4.0, Auditory Research Laboratory, VA Medical Center, Mountain Home, Tennessee. A total of 35 monosyllabic words from the NU No. 6 lists in the presence of multi-speaker babble at different signal (word)-to-noise (babble)- ratios were monaurally presented at 50 dB SL (according to the average of the pure-tone thresholds at 0.5, 1 and 2 kHz). A total of 7 signal-to-noise ratios (SNR, i.e. 0, 4, 8, 12, 16, 20, 24) were used. The participant was asked to repeat back each word as it was heard. The results were calculated based on the signal-to-noise ratio needed to achieve 50% correctly repeated items. The average score between the right and left ears was obtained for analysis purposes ([right ear score (dB SNR) + left ear score (dB SNR)]/2).

##### Pitch pattern sequence [21]

This test was used to evaluate temporal ordering based on pitch differences. Details about the test procedure utilised can be found in Fuente et al. [22]. The average score between the right and left ears was obtained for analysis purposes ([right ear score (%) + left ear score (%)]/2).

##### Dichotic digits [23]

This test is part of the tonal and speech materials for auditory perceptual assessment, Disk 2.0, Auditory Research Laboratory, VA Medical Center, Mountain Home, Tennessee. This task was used to evaluate dichotic listening or binaural integration. Twenty-nine sets of 2 pairs of digits were presented dichotically. Stimuli intensity was set at 50 dB SL according to the average of the pure-tone thresholds at 0.5, 1 and 2 kHz. Participants were instructed to repeat back in a free-recall manner each set of two pairs of numbers. The average score between the right and left ears was obtained for analysis purposes ([right ear score (%) + left ear score (%)]/2).

##### Duration pattern sequence [24]

This test was used to assess temporal ordering based on differences in duration. Forty presentations of sequences of three tone bursts of different duration (250 ms and 500 ms) were used for this task. Stimuli were presented at 50 dB SL based on the pure-tone threshold at 1 kHz. Participants were instructed to name each stimulus of the sequence (e.g. short long short). The average score between the right and left ears was obtained for analysis purposes ([right ear score (%) + left ear score (%)]/2).

##### Adaptive test of temporal resolution (ATTR, Lister et al. [25])

This test was used to evaluate temporal resolution using using both a within-channel and a between-channel gap detection task. The software for this test was installed in a Dell Optiplex 780 desktop computer. The test was run directly from the computer with Bose QuietComfort 15 headphones. For details about the test procedure see Alvarez et al. [26].

### Data analysis

Estimated mean values for each of the hearing outcomes were obtained using multiple linear regression with bootstrapping for the calculation of standard error (10,000 replications). In addition, 95% confidence intervals were obtained through a bias-corrected and accelerated method. All estimations from the regression models were adjusted for age (continuous variable) and level of noise exposure. The latter was categorised into three groups (low, moderate, high), as explained above in the method section. Possible significant differences across jet fuel exposure groups (low, moderate, high) were obtained using a Wald test, controlling for both age (continuous variable) and noise exposure group (low, moderate, high), as explained above in the method section. The statistical analyses were performed with STATA version 14, College Station, Texas, USA. Significant differences were considered at α < 0.05. The statistical power (1- β) was calculated using the G*Power software version 3.1.9.2., Dusseldorf, Germany.

## Results

A total of 107 participants expressed their interest for the study, however contact was lost for 34 of them. For the 73 remaining participants, 2 of them were posted elsewhere, 11 did not have a 2-week minimum period away from the base and 3 of them were excluded after the first initial assessment due to inclusion/exclusion criteria. Therefore, the final sample was comprised of 57 participants. From the 57 participants who were selected and further evaluated, 3 exposure groups were created based on their jet fuel/ chemical exposures (low, moderate, high). The low jet fuel exposure group was comprised of 18 participants, the moderate jet fuel exposure group was comprised of 15 participants and the high jet fuel exposure group was comprised of 24 participants. No significant (*p* > 0.05) age differences were observed across jet fuel exposure groups. Table 1 displays the demographics and job categories for each jet fuel exposure group.

### Hearing thresholds

**Fig. 1:**
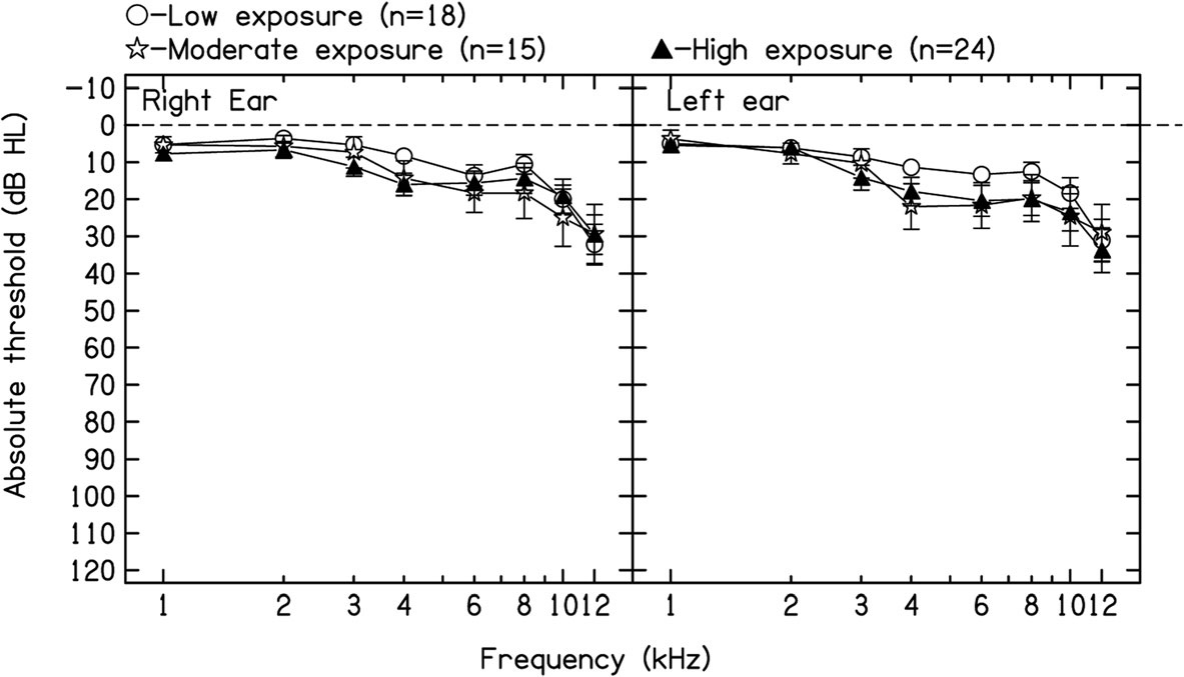
Mean and standard deviation for hearing thresholds (1–12 kHz) for the right and left ears across jet fuel exposure groups. *p < 0.05

**Fig. 2:**
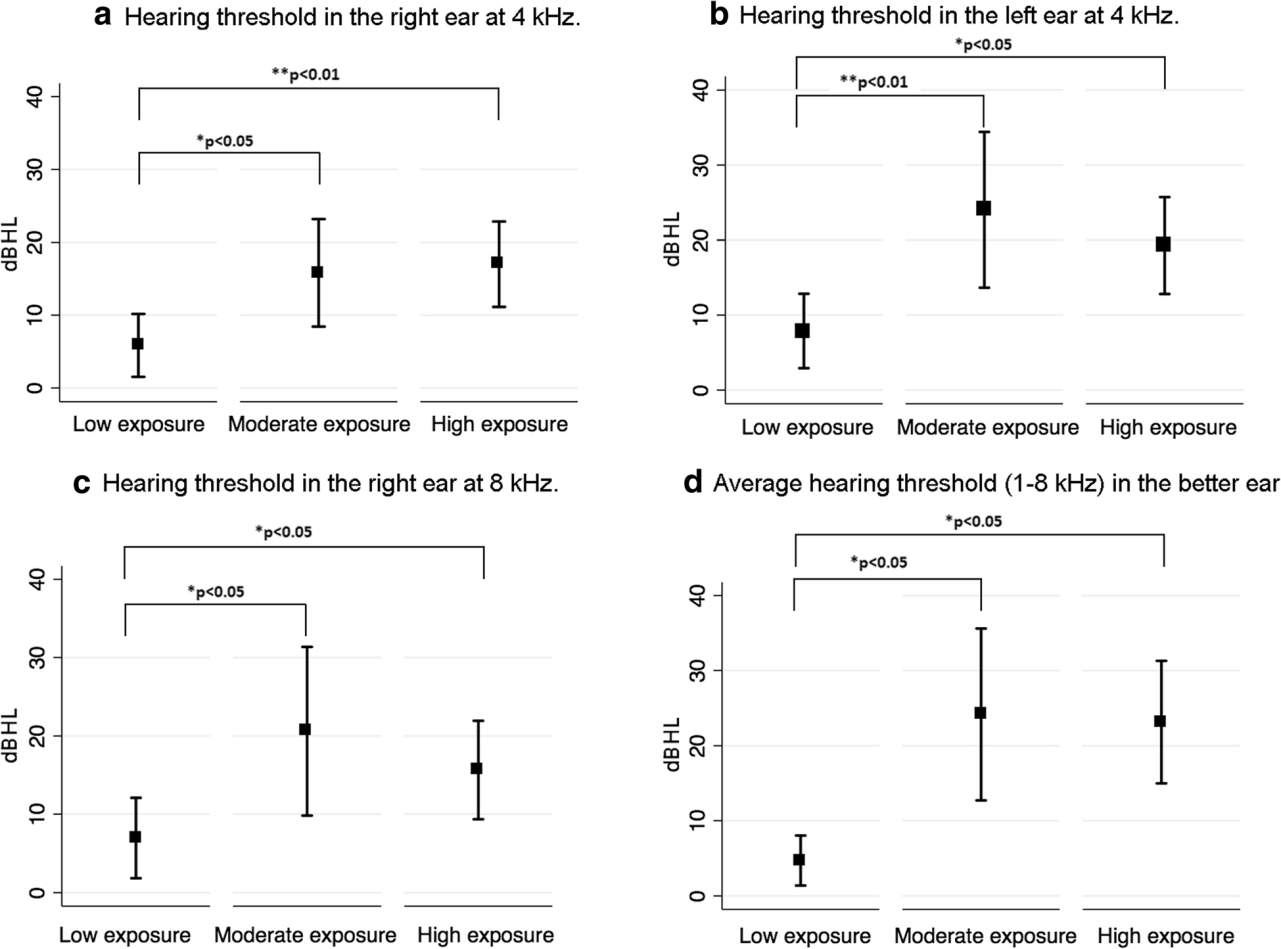
Mean pure-tone thresholds and 95%CI at 4 kHz (right and left ears, panels a and b, respectively), 8 kHz (right ear, panel c) and average hearing threshold (1–8 kHz, panel d) in the better ear for the three exposure groups

Figure 1 displays the mean air conduction pure-tone thresholds (1–12 kHz) for the right and left ears for all three jet fuel exposure groups. Multivariate linear regressions were carried out to estimate the mean for hearing thresholds adjusted for age and noise exposure (i.e. low, moderate, high) using bootstrapping for calculating the standard error (10,000 replications). The *p*-value was estimated through a Wald test. As can be observed in Table 2, a significant association between jet fuel exposure and hearing thresholds was observed for 4 kHz in the right and left ears and for 8 kHz in the right ear. In addition, a significant association between jet fuel exposure and the average hearing threshold across frequencies (1–8 kHz) in the better ear was found. No significant association between jet fuel exposure and the average hearing threshold for ultra-high frequencies in the better ear was found. Further analyses showed that the low jet fuel exposure group presented with significantly lower (i.e. better) hearing thresholds for the aforementioned frequencies and average than groups with moderate and high levels of exposure to jet fuels (see Fig. 2).

**Table 2:** Jet fuel exposure group means for the audiometric hearing thresholds at each tested frequency for the right and left ears and for the average across standard audiometric frequencies (1–8 kHz) and ultra-high frequencies (10 and 12 kHz) for the better ear ^a,b^

Frequency	Low exposure (95% CI)	Moderate exposure (95% CI)	High exposure (95% CI)	*p-value* ^c^
1 kHz				
Right ear	5.51 (1.84–9.19)	7.00 (2.61–11.40)	4.40 (1.89–6.91)	0.521
Left ear	4.40 (0.36–8.43)	4.10 (−0.18–8.37)	5.60 (2.06–9.14)	0.830
2 kHz				
Right ear	2.22 (−1.49–5.93)	6.48 (1.42–11.55)	7.20 (2.78–11.61)	0.295
Left ear	4.91 (1.34–8.50)	8.52 (3.94–13.10)	6.40 (3.17–9.64)	0.525
3 kHz				
Right ear	3.80 (−0.20–7.81)	8.34 (0.72–15.96)	11.73 (7.15–16.30)	0.062
Left ear	6.39 (1.60–11.19)	11.73 (5.37–18.08)	14.96 (9.24–20.68)	0.104
4 kHz				
Right ear	5.85 (1.44–10.25)	15.80 (8.41–23.20)	16.99 (11.05–22.92)	**0.017**
Left ear	7.88 (2.92–12.85)	24.04 (13.66–34.42)	19.27 (12.82–25.72)	**0.007**
6 kHz				
Right ear	11.07 (5.30–16.84)	19.92 (11.29–28.55)	16.54 (10.84–22.25)	0.228
Left ear	10.60 (4.97–16.23)	23.29 (12.09–34.50)	21.45 (13.90–29.01)	0.061
8 kHz				
Right ear	6.98 (1.86–12.10)	20.59 (9.81–31.36)	15.65 (9.35–21.95)	**0.041**
Left ear	9.68 (3.13–16.23)	21.59 (12.16–31.03)	20.91 (13.46–28.36)	0.060
10 kHz				
Right ear	14.18 (6.71–21.66)	28.33 (18.69–37.97)	21.45 (14.67–28.23)	0.085
Left ear	13.18 (5.15–21.20)	27.92 (18.27–37.58)	25.37 (18.32–23.43)	0.038
12 kHz				
Right ear	24.94 (17.29–32.60)	33.58 (25.44–41.72)	32.39 (24.14–40.63)	0.328
Left ear	24.84 (15.43–34.25)	32.91 (24.26–41.57)	36.01 (27.37–44.64)	0.268
Better ear 1–8 kHz	4.70 (1.39–8.01)	12.10 (6.35–17.80)	11.57 (7.49–15.65)	**0.037**
Better ear 10–12 kHz	15.93 (8.48–23.39)	28.97 (20.17–37.78)	25.25 (18.49–32.02)	0.085

^a^Means estimated through a multivariate linear regression using bootstrapping for calculating the standard error (10,000 replications)

^b^Means adjusted by age and noise exposure levels

^c^*p*-value estimated through a Wald test

CI: Confidence interval

### Otoacoustic emissions

**Fig. 3:**
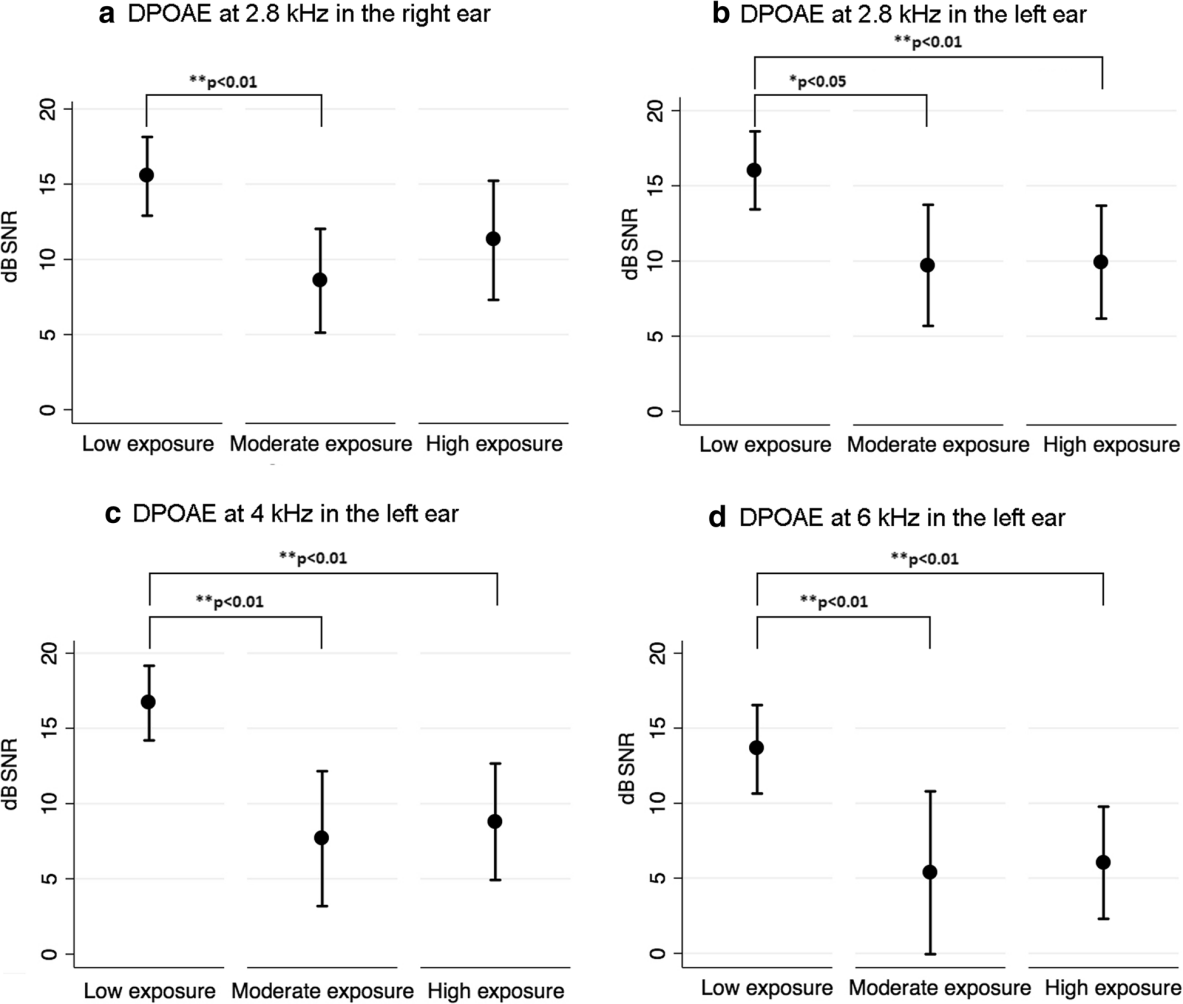
Mean DPOAE amplitudes and 95%CI (dB SNR) at 2.8 kHz (right and left ears, panels a and b, respectively) as well as at 4 (panel c) and 6 kHz (panel d) in the left ear for the three exposure groups

Table 3 displays the mean dB signal-to-noise ratio (SNR) for DPOAEs for the right and left ears across jet fuel exposure groups. Jet fuel exposure was significantly associated with DPOAEs at 2.8 and 6 kHz in the left ear and at 4 kHz in both the right and left ears. Results were controlled for age and noise exposure levels (i.e. low, moderate, high). Figure 3 displays group means for the DPOAEs at frequencies for which jet fuel exposure was significantly associated. As can be observed in Fig. 3, low-exposure participants presented with significantly higher (i.e. better) DPOAE amplitudes than participants with moderate and high exposure levels to jet fuels for 2.8, 4 and 6 kHz in the left ear. In addition, low-exposure participants presented with significantly higher DPOAE amplitudes than moderate-exposure participants at 4 kHz in the right ear.

**Table 3:** Jet fuel exposure group means for DPOAE across frequencies ^a,b^

DPOAE frequency (f2)	Low exposure (95% CI)	Moderate exposure (95% CI)	High exposure (95% CI)	*p*-value^c^
2 kHz.				
Right ear	13.20 (8.93–17.47)	8.11 (3.90–12.32)	10.67 (6.17–15.17)	0.270
Left ear	15.56 (12.13–19.00)	12.97 (9.52–16.42)	12.55 (9.61–15.48)	0.414
2.8 kHz.				
Right ear	15.15 (12.01–18.28)	9.24 (4.65–13.83)	11.79 (8.07–15.51)	0.109
Left ear	16.02 (13.43–18.62)	9.71 (5.68–13.73)	9.92 (6.17–13.67)	**0.004**
4 kHz.				
Right ear	15.52 (12.90–18.14)	8.57 (5.12–12.02)	11.26 (7.31–15.22)	**0.010**
Left ear	16.68 (14.19–19.16)	7.67 (3.18–12.16)	8.79 (4.92–12.67)	**< 0.001**
6 kHz.				
Right ear	12.22 (8.10–16.34)	5.37 (1.25–9.49)	8.60 (4.76–12.43)	0.079
Left ear	13.59 (10.64–16.54)	5.37 (−0.05–10.79)	6.03 (2.28–9.77)	**0.003**
8 kHz.				
Right ear	0.89 (−5.29–7.08)	−7.35 (−12.02– −2.68)	−6.69 (−9.80– −3.58)	0.081
Left ear	−5.31 (−9.80– − 0.82)	−7.68 (−10.99– −4.37)	−7.85 (−10.47– − 5.24)	0.642

^a^Means estimated through a Multivariate Linear Regression using bootstrapping for calculating the standard error (10,000 replications)

^b^Means adjusted by age and noise exposure levels

^c^*p-value* estimated through a Wald test

*CI* Confidence interval

### Auditory brainstem response

**Fig. 4:**
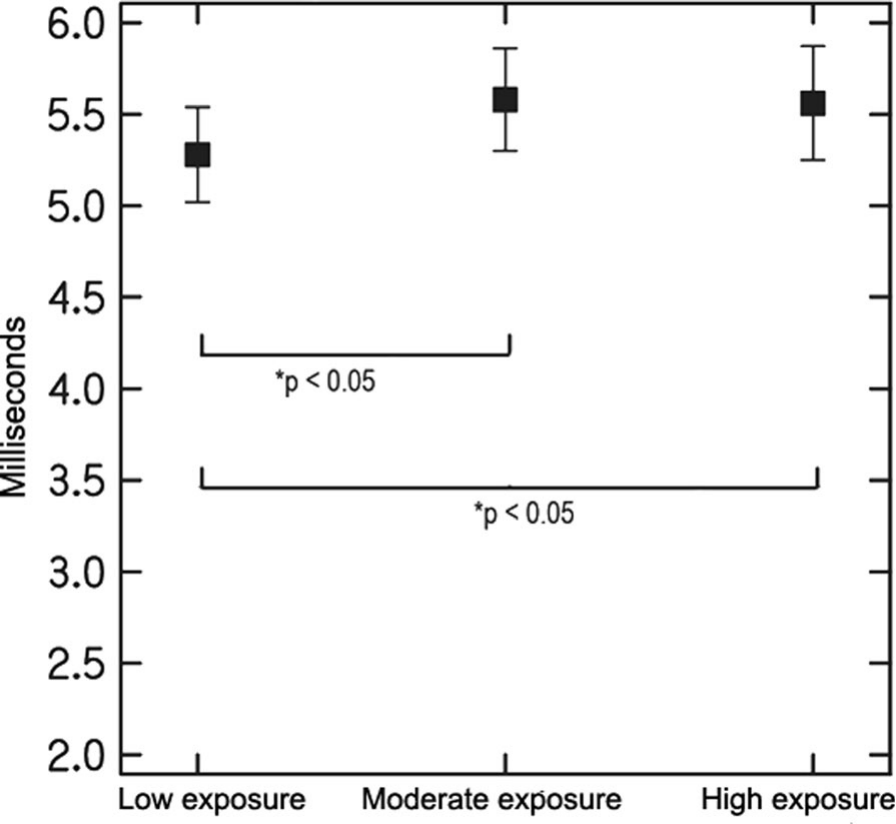
Mean ABR wave V absolute latency and 95%CI in the right ear for the three exposure groups

Table 4 displays means for each jet fuel exposure group for the absolute latencies of I, III and V waves as well as I-III, I-V and III-V IPLs, for both the right and left ears. Jet fuel exposure was significantly associated, controlling for age and noise exposure levels (i.e. low, moderate, high) with the absolute latency of wave V in the right ear. Figure 4 shows that the low jet fuel exposure group presented with significantly shorter latency for wave V than did groups with moderate and high exposure levels to jet fuels.

**Table 4:** Jet fuel exposure group means for ABR absolute latencies (I, III, and V) and inter-peak latencies (I-III, I-V, and III-V) ^a,b^

ABR component	Low exposure (95% CI)	Moderate exposure (95%CI)	High exposure (95%CI)	*p*-value^c^
Wave I				
Right ear	1.47 (1.36–1.59)	1.57 (1.46–1.68)	1.59 (1.52–1.67)	0.288
Left ear	1.46 (1.34–1.57)	1.59 (1.51–1.67)	1.59 (1.53–1.65)	0.142
Wave III				
Right ear	3.65 (3.49–3.80)	3.76 (3.64–3.88)	3.82 (3.73–3.90)	0.257
Left ear	3.59 (3.40–3.78)	3.96 (3.76–4.16)	3.84 (3.72–3.96)	0.073
Wave V				
Right ear	5.08 (4.79–5.37)	5.63 (5.43–5.82)	5.65 (5.44–5.86)	**0.028**
Left ear	5.27 (4.99–5.54)	5.65 (5.40–5.91)	5.55 (5.41–5.69)	0.180
IPL I-V				
Right ear	3.61 (3.34–3.87)	4.06 (3.87–4.26)	4.05 (3.81–4.29)	0.078
Left ear	3.82 (3.58–4.07)	4.06 (3.79–4.33)	3.94 (3.81–4.06)	0.543
IPL I-III				
Right ear	2.17 (2.02–2.32)	2.20 (2.06–2.33)	2.22 (2.11–2.34)	0.886
Left ear	2.15 (1.97–2.33)	2.37 (2.13–2.60)	2.22 (2.11–2.33)	0.400
IPL III-V				
Right ear	1.44 (1.11–1.76)	1.86 (1.60–2.13)	1.83 (1.64–2.02)	0.206
Left ear	1.67 (1.39–1.96)	1.69 (1.49–1.89)	1.71 (1.58–1.85)	0.960

^a^Means estimated through a Multivariate Linear Regression using bootstrapping for calculating the standard error (10,000 replications)

^b^Means adjusted by age and noise exposure levels

^c^*p-value* estimated through a Wald test

*CI* Confidence interval

*IPL* Inter-peak latency

### Behavioural procedures exploring the central auditory nervous system

**Fig. 5:**
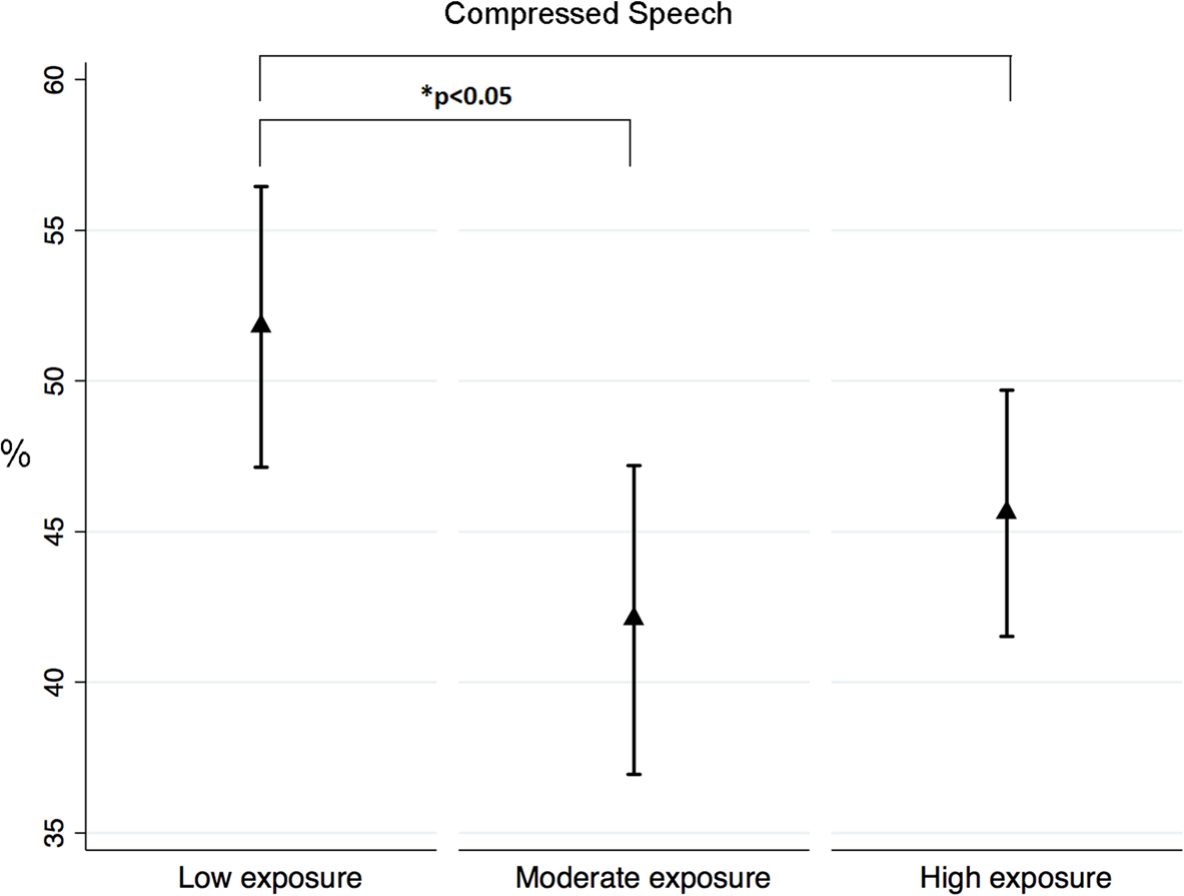
Mean scores and 95%CI for Compressed Speech for the three exposure groups

**Fig. 6:**
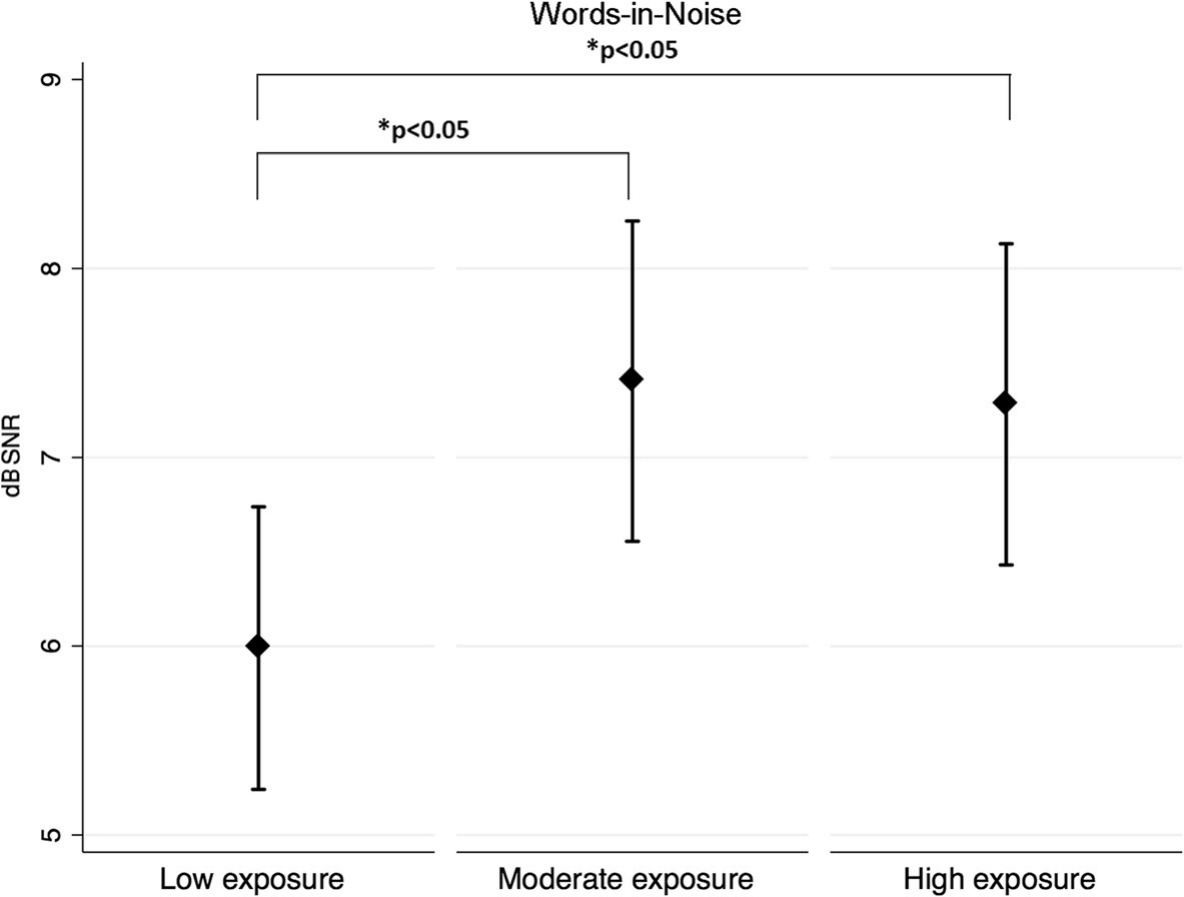
Mean Scores and 95%CI for Words-in-Noise for the three exposure groups

Table 5 displays the mean scores for the behavioural tests assessing the central auditory nervous system. Jet fuel exposure was significantly associated with compressed speech and words-in-noise test scores. Results were controlled for age and noise exposure levels (i.e. low, moderate, high). Further analyses showed that the low jet fuel exposure group presented with significantly better test scores for both the compressed speech and words-in-noise tests than the moderate jet fuel exposure group. In addition, the low jet fuel exposure group presented with significantly better scores for words-in-noise than the high jet fuel exposure group (see Figs. 5 and 6).

**Table 5:** Jet fuel exposure group means for behavioural procedures investigating central auditory functions ^a,b^

Test	Low exposure (95% CI)	Medium exposure (95% CI)	High exposure (95% CI)	*p-value* ^c^
Compressed speech, %	51.80 (47.14–56.46)	42.07 (36.95–47.20)	45.60 (41.52–49.69)	0.039*
Words-in-noise, dB SNR	5.99 (5.24–6.74)	7.40 (6.55–8.25)	7.28 (6.43–8.13)	0.038*
Dichotic digits, %	92.67 (87.80–97.54)	89.69 (87.08–92.31)	87.23 (81.93–92.53)	0.413
Pitch pattern sequence, %	100.0 (98.16–100.0)	97.28 (94.50–100.0)	94.13 (89.97–98.30)	0.082
Duration pattern sequence, %	99.50 (98.08–100.0)	98.42 (97.00–99.84)	96.82 (93.53–100.0)	0.434
ATTR, ms				
Within channel	3.51 (2.31–4.71)	3.28 (2.46–4.10)	5.24 (2.91–7.56)	0.228
Across channel	48.63 (34.90–62.35)	41.65 (29.40–53.90)	46.53 (33.98–59.09)	0.720

^a^Means estimated through a quantile regression using bootstrapping for calculating the standard error (10,000 replications)

^b^Means adjusted by age and noise exposure levels

^c^*p-value* estimated through a Wald test

*CI*: Confidence interval

*ATTR* Auditory test of temporal resolution

*dB SNR* dB signal-to-noise ratio

*Ms* Milliseconds

Values for compressed speech, words-in-noise, pitch pattern sequence, and duration pattern sequence represent the average score between the right and left ears

## Discussion

### Effects of jet fuel exposure on hearing thresholds

Participants with moderate and high exposure levels to jet fuels presented with significantly worse hearing thresholds at 4 kHz in both ears and at 8 kHz in the right ear. In addition, the multivariate regression model adjusted by age and noise exposure levels (i.e. low, moderate, high) showed that jet fuel exposure was significantly associated with the average hearing threshold across frequencies (1–8 kHz) in the better ear. These results are in agreement with a previous study on the RAAF personnel by Guest et al. [27], who reported that the hearing thresholds of fuel talk maintenance workers were worse than expected by ISO 1999 population databases. In addition, Kaufman et al. [5] found that jet fuel exposure in military workers increased the adjusted odds of a 15 dB or greater permanent hearing loss when combined with noise exposure during the first 12 years of exposure. It should be noted, however, that in the present study, most of the participants presented with normal hearing thresholds (i.e. equal to or better than 20 dB HL), and their mean tenure in the defence sector was 17.8 years. Also, the results from the present study are in agreement with Prasher et al. [28]. Those authors found a significant effect of group category (i.e. civilian aircraft maintenance workers) on pure-tone thresholds as compared to both workers exposed to chemicals only and workers without exposure to either noise or chemicals. However, contradictory results were found by Hughes and Hunting [29]. They carried out a longitudinal study of a group of civilian and military aviation personnel. The authors investigated changes in pure-tone thresholds at 2, 3 and 4 kHz over a 7-year period and how changes were associated with variables such as age at first audiogram, noise exposure and exposure to chemicals, including organic solvents and JP-8. The authors found that hearing loss (i.e. a change in pure-tone threshold equal to or higher than 10 dB HL during the study period) was associated with age at first study audiogram, length of follow-up time and noise exposure. No additional risk for hearing loss among personnel exposed to either noise and chemicals or chemicals only was found.

The differences in results between Hughes and Hunting [29] and the present study may be due to the methodological differences. In this study, we did not classify participants based on their hearing thresholds. Instead, we compared mean hearing thresholds across three jet fuel exposure groups in a cross-sectional manner. Hughes and Hunting categorised participants based on changes in hearing thresholds, and participants included both full-time and part-time aviation personnel. In addition, the follow-up period was not the same for all participants. Audiograms for some participants were separated by 6 years and only 1 year for others. Thus, the time participants were exposed to chemicals including JP-8 may not have been long enough to observe a change in audiometric thresholds. For example, the average follow-up for participants exposed only to chemicals was 1.8 years. Based on the results of the present study, we conclude that jet fuel exposure combined with noise exposure can have an adverse effect on pure-tone thresholds mainly at high frequencies.

### Effects of jet fuel exposure on OHC function

The results from DPOAE showed that jet fuel exposure has an adverse effect on DPOAE amplitudes (SNR) at 2.8, 4 and 6 kHz in both ears. These results are not in agreement with Prasher et al. [28], who did not find an effect of exposure to chemicals, including jet fuels, and noise on DPOAE amplitudes in civilian aircraft maintenance workers. DPOAE results found in the present study suggest that higher hearing thresholds at 4 kHz observed in participants with high exposure levels to jet fuels are associated with OHC dysfunction. This hypothesis can also be supported by the ABR results. Prolonged wave V latencies were found in participants with high exposure levels to jet fuels as compared to participants with low exposure levels. This finding is expected in individuals with poorer audibility at high frequencies, as was the case for participants with high levels of exposure to jet fuel as compared to participants with low levels of exposure to jet fuel.

### Effects of jet fuel exposure on the central auditory system

In this study, the central auditory nervous system was explored using both behavioural and electrophysiological techniques. Regarding the former, jet fuel exposure was significantly associated with compressed speech and words-in-noise test scores. For both procedures, controlling for age and noise exposure levels (i.e. low, moderate, high), participants with low levels of exposure to jet fuel presented with significantly better results than participants with moderate and high exposure levels. These results are in line with the results investigating pure-tone thresholds and OHC function (DPOAEs). No significant effect of jet fuel exposure on temporal patterning (i.e. pitch pattern sequence and duration pattern sequence), temporal resolution (i.e. ATTR) and binaural integration (i.e. dichotic digits) was found. It is important to mention that for the pitch pattern sequence test, the effect size associated with jet fuel exposure had a power of 55%. The minimum power is 80%, thus due to the sample size, it is not possible to exclude an effect of jet fuel exposure on PPS test results. In addition, the electrophysiological procedure (i.e. ABR) did not show an effect of jet fuel exposure on the conduction of auditory information at the brainstem level. The only effect of jet fuel exposure was found on wave V latency in the right ear. These results are different than the findings reported by Prasher et al. [28] for a group of aircraft maintenance workers. The authors found that 32% of these workers exposed to chemicals, including jet fuels, and noise presented with prolonged ABR inter-peak latencies. As explained above, wave V latency is expected to be delayed in the presence of poorer sound detection abilities, which was the case among participants with high levels of exposure to jet fuel, who also showed a significantly longer wave V latency than the other two jet fuel exposure groups.

We hypothesise that the observed effect of jet fuel exposure on compressed speech and words-in-noise tests was associated with OHC dysfunction rather than central auditory nervous system dysfunction. This is because OHC dysfunction relates to a decrement in frequency selectivity and thus the person’s capacity to process frequency differences among sounds. This is closely associated with speech perception in challenging conditions, as is the case with both compressed speech and words-in-noise tests.

### Limitations of the study

This study set out to determine if an association existed between the exposure to jet fuel and noise and auditory functions of workers on a base of the RAAF. The information obtained through an expert examination of industrial hygiene records, a review of historic industrial hygiene records of the studied RAAF base, and an interview with each participant allowed for the classification of the participants in exposure groups ranging from low to high. This was done separately for jet fuel and noise exposures. The information gathered, however, was insufficient to permit the reconstruction of the lifetime exposure history of the participants, as this workforce rotates among bases, and with each assignment, work conditions, schedules and responsibilities vary. In addition, the cross-sectional design did not permit a longitudinal analysis of the hearing status of this population. These were significant limitations of the study. In addition, the levels of exposures to noise and jet fuel were not independent, and higher levels of exposure to noise usually occurred in jobs that also involved higher levels of exposure to fuels. High levels of noise exposure occurred occasionally, and in those instances, the use of hearing protection was required for those exposed. In addition, classifications to jet fuels and noise were based on retrospective environmental (group) data and subjective rather than objective criteria such as solvent biomarkers and/or airborne concentrations to jet fuels/solvents and noise dosimetry. The possibility of bias in the classification of exposure groups cannot be ruled out. Lastly, it is possible that participation was greater among those who have experienced hearing difficulties in daily life, which could have biased our results against the null hypothesis. Nevertheless, the results of the audiological tests conducted were able to detect a difference in performance between workers who were the least exposed and those whose levels of exposure to fuels were higher.

## Conclusions

The present study found a chronic effect of jet fuel exposure on pure-tone thresholds, DPOAE amplitudes, ABR wave V latency, and scores for both compressed speech and words-in-noise. Air Force personnel exposed to low levels of jet fuels presented with significantly better results for the aforementioned hearing tests than personnel with moderate and high levels of exposure to jet fuels. These results suggest a peripheral auditory dysfunction associated with jet fuel exposure in humans. No evidence of chronic central auditory nervous system dysfunction associated with jet fuel exposure was found in this study, however further research is required to explore possible chronic adverse effects of jet fuel exposure on the central auditory nervous system in humans.

## Data Availability

The dataset used and analysed during the current study are available from the corresponding author on reasonable request.

## Abbreviations

6SQN: Number 6 Squadron
ABR: Auditory brainstem response
ATTR: Auditory test of temporal resolution
dB: Decibel
DPOAE: Distortion product otoacoustic emissions
HL: Hearing level
IPL: Inter-peak latency for auditory brainstem responses
ISO: International Organization for Standardization
LAeq: A-weighted Equivalent Sound Level
MEOMS: Mechanical Equipment Operations Management Systems
OHC: Outer hair cell
RAAF: Royal Australian Air Force
SL: Sensation level
SNR: Signal-to-noise ratio
